# Prevalence, awareness, treatment, and control of hypertension in Cameroonians aged 50 years and older: A community‐based study

**DOI:** 10.1002/hsr2.44

**Published:** 2018-04-27

**Authors:** Frank L. Tianyi, Valirie N. Agbor, Alfred K. Njamnshi

**Affiliations:** ^1^ Mayo‐Darlé Sub‐divisional Hospital Banyo Adamawa Region Cameroon; ^2^ Ibal Sub‐divisional Hospital Oku North‐West Region Cameroon; ^3^ Faculty of Medicine and Biomedical Sciences The University of Yaoundé I Yaoundé Cameroon; ^4^ Brain Research Africa Initiative (BRAIN) Yaounde Cameroon

**Keywords:** awareness, community‐based, control, elderly, hypertension, obesity, overweight, treatment

## Abstract

**Aims:**

To assess the prevalence of hypertension (HTN) in a rural elderly population (50 y and older) in Cameroon; evaluate the rates of awareness, treatment, and control of HTN in this population; and describe factors associated with HTN in this population.

**Methods and Results:**

A total of 501 participants aged 50 years and older were randomly recruited from May to July 2013 in a house‐to‐house survey of the Batibo Health District. Data were measured using standardized methods modelled after the World Health Organization STEPwise approach to Surveillance. The Statistical Package for the Social Sciences version 20.0 was used for statistical analysis. Chi‐square, Fisher's exact or Student *T* test were used to compare variables. A multivariable logistic regression analysis was used to identify factors associated with HTN in this population. In our study population, 31% of the participants were men, with a mean age of 65.4 ± 8 years; women had a mean age of 61.4 ± 9 years. The prevalence of HTN was 57.3% (95% CI, 52.9‐61.6). The awareness rate was 63.4%, treatment rate 96.7%, and control rate 32.4%. Being overweight/obese was independently associated with HTN in this group (odds ratio = 3.46; 95% CI, 2.38‐5.03; *P* < .001).

**Conclusion:**

The prevalence of HTN amongst the elderly in the Batibo Health District is high. Emphasis should be on patient education to improve the rates of blood pressure control amongst patients on treatment for HTN. Healthy lifestyle measures such as reduction in salt intake and increase in physical exercise should be encouraged amongst the elderly.

## 1 INTRODUCTION

Hypertension (HTN) is a major risk factor of cardiovascular and cerebrovascular diseases and is associated with a high degree of morbidity and mortality, especially in the elderly.^1^ Hypertension accounts for approximately 1 million deaths amongst the 1 billion adults living with HTN worldwide.^2^, ^3^ The number of persons living with HTN globally is expected to rise to about 1.5 billion by the year 2025.^2^, ^3^ Sub‐Saharan Africa (SSA) bears a great burden of HTN, which is the leading cause of heart failure^4^, ^5^ and stroke^6^ and accounts for over 80% of all cardiovascular disease‐related deaths.^3^, ^7^ In Cameroon, the prevalence of HTN increased over 5‐fold between 1994 and 2003,^8^ with a recent community‐based study estimating the prevalence of HTN in the general population at 29.7%.^7^ Elderly populations harbour the highest prevalence of HTN,^3^, ^7^, ^9^ owing to a strong positive correlation between increasing age and HTN.^2^, ^3^

Globally, the annual increase in the elderly population is greater (1.9%) than that in the total population (1.2%).^10^ In fact, it is projected that by 2050, the number of individuals older than 60 years will be approximately 2 billion and will account for about 22% of the world's population, four‐fifths of whom will reside in developing countries in Africa, Asia, or Latin America.^10^ In Cameroon, the population aged 65 years and older currently represents 3.5% of the total population.^11^ With a projected increase in the proportion of elderly individuals, the description of the health profile amongst this population is vital for planning and implementing policies related to a healthy old age.

The traditional cut‐off to define an elderly population is 65 years. This age marks a decreased active contribution to society and is most often the age at which one can begin to receive pension benefits.^12^ This cut‐off to define the elderly, however, is more applicable to high‐income countries, where there is a relatively high life expectancy. The average life expectancy in Cameroon is 54 years.^11^ Considering this fact, we designed our study to include people aged 50 years and older; this includes a considerable number of the “elderly” in our community, most of whom would have been missed if the cut‐off of 65 years had been used. Furthermore, our cut‐off of 50 years correlates with the MDS (Minimum Data Set) Project collaborators at the 2000 Harare MDS Workshop, who initially chose 60 years as the definition for an elderly population,^13^ but after realizing that this value failed to take into account the situation of older persons in SSA, they changed the definition to 50 years.^13^, ^14^ This proved to be a better representation of the elderly in SSA.^14^

Mindful of this, we sought to assess the prevalence and factors associated with HTN in a rural elderly Cameroonian population aged 50 years and above and to evaluate the rates of awareness, treatment, and control of HTN in the same population.

## 2 METHODS

### 2.1 Study setting and duration

This was a community‐based, cross‐sectional and analytic study performed from May to July 2013 in 9 health areas (Ashong, Batibo urban, Ewai, Ewoh, Gwofon, Guzang, Kugwe, Kulabei, and Tiben) in the Batibo Health District (BHD). Batibo is a rural community in the Momo division, North‐West Region, Cameroon. Batibo Health District had an estimated population of 78 972 inhabitants in 2012. The BHD covers all of Batibo and Widikum subdivisions. It is made up of 14 health areas and 22 health units, with a district hospital, and covers a surface area of about 587 km^2^, and a population density of 135 inhabitants per square kilometre.

### 2.2 Study population and sampling

Eligible participants aged 50 years and older were recruited from each health area using a simple random sampling technique. Participants who refused to sign an informed consent form or provide a verbal consent were excluded from the study.

Sample size was determined using the following formula:

$$n=\frac{Z^{2}\;P\;\left(1-P\right)}{d^{2}},$$

where *n* is the sample size (number of elderly participants), *P* is the expected prevalence of HTN in an elderly population (*P* = .58),^15^ and *d* is the precision (if 5%, *d* = 0.05). *Z* statistics (*Z*): For the level of confidence of 95%, which is conventional, *Z* value is 1.96 for a 95% CI.

A minimum of 375 elderly participants were required for this study.

### 2.3 Data collection

Data were collected through predesigned questionnaires adapted from the World Health Organization (WHO) STEPwise approach to Surveillance (STEPS) in 3 steps. In step 1, an interviewer‐administered face‐to‐face questionnaire was used to obtain participants' demographic information. We collected data on age, sex, marital status, educational level, and occupational level. We also used these questionnaires to evaluate awareness and treatment of HTN. In step 2, we measured blood pressure (BP) using a standardized protocol, with the participant in a seated position and after at least 10 minutes' rest, with a manual BP measuring device that covered at least 80% of the arm, and a stethoscope. The Korotkoff sounds of phases I and V were recorded as systolic and diastolic BP (SBP and DBP), respectively. The mean of 2 measures performed at least 5 minutes apart was used for all analyses. In step 3, height was measured with a calibrated stadiometer to the nearest 0.5 cm. Weight was also measured with the patients in light clothes, using a scale balanced to the nearest 0.1 kg.

### 2.4 Definitions

1. For the purpose of this study, HTN was defined as SBP ≥ 140 and/or DBP ≥ 90 mmHg, or report of current use of antihypertensive medication.^16^
2. An elderly was defined in our study as being 50 years old or older.
3. Hypertension awareness rate was defined as the proportion of individuals with HTN who affirms either having been diagnosed with HTN by a health professional and/or taking medication for HTN.
4. Hypertension treatment rate was defined as the proportion of hypertensive individuals who were aware of their hypertensive status and reported taking medication for HTN.
5. Hypertension control was defined as the proportion of individuals on treatment for HTN (pharmacotherapy, or lifestyle modifications, or both) with SBP < 140 mmHg and DBP < 90 mmHg.
6. Occupational level was categorized into low (no technical know‐how or expert training required, eg, manual workers), medium (requires a degree of technical know‐how but no expert training, eg, salesmen, and bike and taxi drivers), and high (major professionals requiring advanced training, eg, teachers, health personnel, and accountants).
7. Formal educational level was categorized as none (no formal education), low (primary education), medium (secondary education), and high (university education).
8. Body mass index (BMI) was calculated as weight in kilograms divided by the square of the height in metres, and BMI‐based body habitus (in kg/m^2^) was classified as underweight (BMI < 18.5), normal weight (BMI = 18.5‐24.9), overweight (BMI = 25.0‐29.9), and obese (BMI ≥ 30).^17^

### 2.5 Data analysis

Data were entered into and analysed using the Statistical Package for the Social Sciences version 20.0. Qualitative variables were reported as frequencies or proportions, while quantitative variables were reported as means alongside their corresponding standard deviations. Quantitative variables were compared using the Student *t* test, whereas the Chi‐squared or Fisher's exact test was used to compare categorical variables. To account for confounders, potential risk factors (age, sex, educational level, and occupational level) with *P* values <.25 in bivariate analysis were further assessed using a multivariable logistic regression analysis according to the method suggested by Bursac et al.^18^ A 2‐tailed statistical significance was set at a *P* value below .05.

### 2.6 Ethical considerations

Ethical approval to conduct the study was obtained from the Institutional Review Board of the Faculty of Health Sciences, University of Buea, Cameroon. Apart from the inconvenience of taking time to answer the research questionnaire, participants were not exposed to any undue risk. The participants had their BPs measured for free, and they received free advice on lifestyle modifications to prevent or treat HTN. A translator was used for participants who did not understand English, French, or the local lingua franca. All participants provided a verbal consent to participate in the study.

## 3 RESULTS

In total, 501 individuals participated in this study, 68.8% of whom were women. Table 1 depicts the baseline socio‐demographic characteristics of our study population. The mean age for the entire population was 62.7 ± 9, and their ages spanned from 50 to 110 years. Men (mean age = 65.4 ± 8 y) were older than women (61.4 ± 9 y). There was a significantly greater proportion of single women, compared with men, in our study (59.7% vs 10.3%; *P* < .001, Student *t* test). There was a high rate of illiteracy, with 82% of participants having no formal education. However, more men, compared with women, had formal education. Also, a greater majority of men, compared with women, had at least a medium‐level occupational level.

**Table 1:** Baseline sociodemographic characteristics of the study, Batibo Health District, May to June 2013

Characteristics	Category	Male (%)	Female (%)	Total (%)	*P* Value
Age group, y	Mean ± SD	65.4 ± 8	61.4 ± 9		<.001^a^^,^ b
	50‐59	37 (23.7)	158 (45.8)	195 (38.9)	.031^a^^,^ c
	60‐69	64 (41.0)	121 (35.1)	185 (36.9)	.825^c^
	70‐79	44 (28.2)	47 (13.6)	91 (18.2)	.267^c^
	≥80	11 (7.1)	19 (5.5)	30 (6.0)	.149^c^
Marital status	Single	16 (10.3)	206 (59.7)	222 (44.3)	<.001^a^^,^ d
	Married	140 (89.7)	139 (40.3)	279 (55.7)	.952^d^
Occupational level	Low	114 (73.0)	334 (96.8)	448 (89.4)	.428^c^
	Medium	38 (24.4)	9 (2.6)	47 (9.4)	.043^a^^,^ c
	High	4 (2.6)	2 (0.6)	6 (1.2)	.952^c^
Educational level	None	99 (63.5)	312 (90.4)	411 (82.0)	.097^c^
	Primary	35 (22.4)	26 (7.5)	61 (12.2)	.007^a^^,^ c
	Secondary	16 (10.3)	6 (1.8)	22 (4.4)	.178^c^
	University	6 (3.8)	1 (0.3)	7(1.4)	.492^c^
Weight, kg	Mean ± SD	66.2 ± 10	63.7 ± 13		.026^b^
Height, m	Mean ± SD	1.6 ± 0	1.5 ± 0		.475^b^
BMI, kg/m^2^	Mean ± SD	25.6 ± 6	25.8 ± 4		.595^b^
SBP, mmHg	Mean ± SD	136.8 ± 20	133.3 ± 22		.096^b^
DBP, mmHg	Mean ± SD	83.9 ± 13	84.7 ± 15		.554^b^

Abbreviations: BMI, body mass index; DBP, diastolic blood pressure; SBP, systolic blood pressure; SD, standard deviation.

a Statistically significant variable.

b Student *t* test–derived *P* value.

c Chi‐square–derived *P* value.

d Fisher exact–derived *P* value.

Of the 501 participants, 287 were hypertensive, giving a prevalence of 57.3% (95% CI, 52.9‐61.6). A higher proportion of men had HTN, compared with women (60.9% vs 55.7%; Table 2).

**Table 2:** Prevalence of hypertension stratified according to age group and sex, Batibo Health District, May to June 2013

Age Group, y	Male with Hypertension		Female with Hypertension		Total Participants with Hypertension	
	N	%	N	%	N	%
50‐59	22	59.5	85	53.8	107	54.9
60‐69	44	68.6	75	62.0	119	62.2
70‐79	23	52.3	23	48.9	46	50.5
>80	6	54.5	9	30.0	15	50
Total	95	60.9	192	55.7	287	57.3

Abbreviation: N, frequency.

After multivariable logistic regression analysis, elevated BMI (overweight/obesity) was the lone factor independently associated with HTN amongst elderly persons in this rural community (adjusted odds ratio = 3.46; 95% CI, 2.38‐5.03; *P* < .001, χ^2^ test; Table 3).

**Table 3:** Factors associated with hypertension on multivariable logistic regression analysis, Batibo Health District, May to June 2013

Variables		OR	95% CI	*P* Value^a^	Adjusted OR	95% CI	*P* Value^a^
Age, y^b^	50‐69	1.14	0.96‐2.18	.08			
	>70						
Sex (male)^b^		1.24	0.84‐1.83	.272			
Marital status (single)		1.07	0.75‐1.53	.693			
Illiterate (yes)^b^		1.36	0.85‐2.18	.201			
Occupational level (≥medium)^b^		0.55	0.30‐0.98	.045^c^	0.56	0.23‐1.32	.183
Overweight/obesity (yes)^b^		5.42	2.44‐5.13	<.001^c^	3.46	2.38‐5.03	<.001^c^

Abbreviation: OR, odds ratio.

a Two‐tailed *P* values, generated using the Fisher exact test.

b Included in the multivariable analysis.

c Significant *P* value.

**Figure 1:**
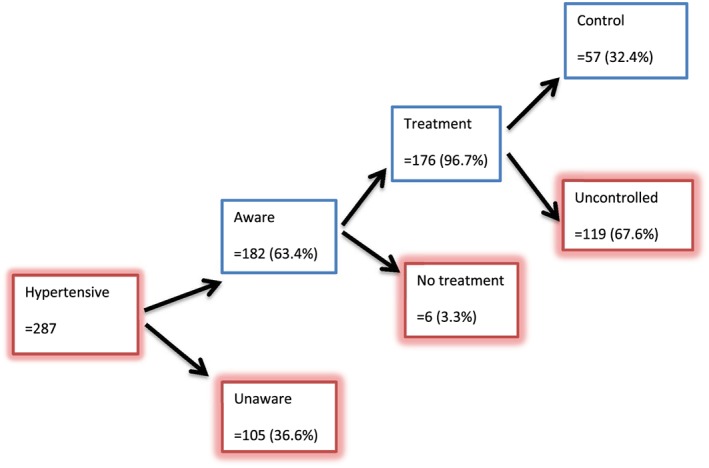
Awareness, treatment, and control rate of hypertension in elderly in the Batibo Health District

Of the 287 participants with HTN in our study, 182 (63.4%) had prior knowledge of having HTN, amongst which 176 (96.7%) were on treatment and 57 (32.4%) had a controlled BP (Figure 1).

## 4 DISCUSSION

We sought to evaluate the prevalence and factors associated with HTN in a rural elderly Cameroonian population. In addition, we aimed to determine the awareness, treatment, and control rates of HTN in the same population. The prevalence of HTN in our study population was 57.3%. Of the participants with HTN, 63.4% were aware of their condition. Of those who were aware of their hypertensive status, 96.7% were on at least 1 antihypertensive agent, 32.4% of whom had a controlled BP. Elevated BMI (overweight/obesity) was the only factor that was independently associated with HTN in our study population.

The 57.3% prevalence rate of HTN in this study is similar to rates of 47.5% reported by Dzudie et al^19^ in an earlier study in Cameroon. Our finding is higher than the prevalence of 37.8% reported by Lemogoum et al^20^ in the far North Region of Cameroon and is almost twice the prevalence of 24.6% reported 15 years earlier by Kamadjeu et al.^21^ Also, our finding is consistent with rates of 52.9%,^15^ 57.1%,^15^ 58.2%,^22^ and 51.8%^23^ obtained from other developing countries. However, this finding was much higher than reports from other SSA countries like Malawi, Rwanda, and Tanzania (36.6%‐41%),^24^ and elsewhere.^25^, ^26^ The value we obtained is lower than the values in reports from Senegal (64.5%),^27^ Zimbabwe (72.4%),^28^ and South Africa (77.9%)^15^ and the 74.1% to 89% prevalence in high‐income countries.^29^, ^30^ These differences could be due to heterogeneity in sampling methods and cut‐off age for the elderly population, ranging from 35 to 74 years across various studies.^1^, ^15^, ^26^, ^27^, ^30^, ^31^, ^32^, ^33^

Compared with younger adults, older individuals are at least twice more likely to develop HTN.^33^ With age, arteries dilate and stiffen, neurohormonal mechanisms such as the renin‐angiotensin‐aldosterone system decline, and there is a progressive development of renal glomerulosclerosis and interstitial fibrosis, which is associated with a decline in glomerular filtration rate and reduction of other homeostatic mechanisms,^1^, ^34^, ^35^, ^36^ altogether accounting for an increased prevalence of HTN in elderly populations. The prevalence of HTN in the general population in Cameroon has been on the rise over the past decades, from 16.4% in 1998^37^ to 29.7% in 2015.^7^ This is in coherence with global trends, and recent estimates project a further increase in these values.^38^ With the rising proportion of elderly populations especially in low‐ and middle‐income countries (LMICs), our findings, amongst others studies,^15^, ^25^, ^26^, ^33^ highlight the burden of HTN in elderly populations in Cameroon and the need to devise and implement strategies to curb this rising prevalence.

After a multivariable logistic regression analysis, being overweight/obese was the only factor that was independently associated with HTN in our study population. This is in line with findings in Central Africa,^39^ Costa Rica,^40^ Senegal,^27^ and other LMICs.^15^ The association between HTN and overweight/obesity is well‐recognized and has been described by several authors,^15^, ^33^, ^41^, ^42^, ^43^ even though the exact mechanism behind the relationship is poorly understood. Complex interactions between metabolic and neurohormonal pathways, with resultant alterations in insulin resistance, the renin‐angiotensin‐aldosterone system, and sympathetic tone, could explain the occurrence of HTN amongst people who are overweight/obese.^44^, ^45^, ^46^, ^47^, ^48^, ^49^

The awareness and treatment rates of 63.4% and 96.7% in our study are similar to the 62.4% and 93.3% rates in Pakistan, and the 69% and 90.8% rates in Peru, reported in a review of HTN in a slightly younger population in 9 LMIC by Irazola et al.^31^ Our awareness rates were higher than the 44.6%^22^ and 44.9%^23^ found in the elderly in Mexico and India, respectively, and the 48.3%^15^ found by the WHO's Study on Global Aging and Adult Health across LMICs. The value was lower than rates reported in high‐income countries.^30^, ^32^

Our control rates were significantly lower than the 46% and 71% obtained in Pakistan and Peru, respectively, in a review of HTN in a slightly younger population in 9 LMIC by Irazola et al.^31^ Our rates were considerably higher than those found in an elderly population in other LMICs.^15^, ^22^, ^23^

A high rate of awareness and treatment of HTN was noted in this study population. This could be due to the fact that a few years prior to our study, the staff of the BHD organized free consultation days with free screening for diabetes and HTN, after which they launched an HTN and diabetes clinic that ran every last Thursday of the month, at the hospital. All patients had to do was turn up each month at the HTN clinic and get free BP measurements and purchase a refill of their antihypertensive medications. However, despite the high rates of awareness and treatment, the control rates remained low. The Pan‐African Society of Cardiology recently identified roadblocks against the effective control of HTN in Africa, amongst which were lack of established national policies for controlling HTN, low prioritization of noncommunicable diseases with a reluctance to implement policies on noncommunicable diseases by government officials, shortage of health care professionals (physicians, nurses, and trained health workers) at primary care level with very low physician/patient ratio, lack of quality and affordable anti‐HTN medications, low mastery of the effects of HTN and its consequences by health care professionals and patients alike, poor patient education with a resultant difficulty in changing lifestyles, and false health beliefs that HTN is curable.^50^ Effectively addressing these roadblocks could improve the treatment and control rates of HTN in Africa in general, and in Cameroon in particular. Medication nonadherence amongst patients with HTN has also been proposed as a cause of poor control rates, with a recent study reporting a nonadherence rate as high as 66.7%.^51^ Furthermore, HTN has been associated with the development of cognitive impairment, which could explain failure to take antihypertensive medication, and account for the poor adherence to antihypertensive medication and the resulting poor control rates.^52^ Proposed solutions to these problems include adopting and implementing national guidelines and policies for the effective detection, treatment, and control of HTN; recognizing HTN as a public health priority and allocating appropriate resources for effective detection, treatment, and control of HTN; designing and implementing training courses for community health workers and health care personnel to improve the quality of anti‐HTN treatment; improving access to affordable and high‐quality antihypertensive treatment; improving patient education on the consequences of HTN; and investing in population‐level interventions for preventing HTN such as reducing salt intake and obesity levels, increasing fruit and vegetable intake, and encouraging physical exercise.^50^ In addition, home BP monitoring has been suggested as a potential strategy to improve treatment compliance and, consequently, optimal BP control.^53^ Hence, home BP monitoring could serve as a valuable tool in reducing the burden of HTN in LMICs.^53^, ^54^

In response to the rising burden of HTN in Cameroon, the Ministry of Public Health developed the “national strategy for hypertension and diabetes control” and the “development of training and task‐shifting programmes to improve detection and management at the primary care level.”^19^, ^55^, ^56^, ^57^ The aim was to promote equitable access to quality health services in order to reduce the morbidity and mortality associated with this condition.^7^, ^58^ Nonetheless, these programmes remain effective mainly at the tertiary levels, with very few of such programmes existing at the primary care level. From our experience, the success of such programmes at the primary care level often relies on the creativity and interest of the local health authorities in addressing these concerns. From this study, we argue that initiatives such as free consultation days for the elderly could improve detection of HTN in primary care settings, and the creation of a HTN clinic could facilitate their follow‐up and improve treatment and control rates. We recommend that such initiatives be implemented throughout the national territory to provide a lasting solution to the rising HTN burden.

### 4.1 Study limitations

The findings from this study should be interpreted considering its limitations. The study was cross‐sectional, meaning HTN was defined after measurements from a single encounter. Hypertension should normally be confirmed on repeated measurements.^59^ Hence, participants with episodic elevation of BP above 140/90 mmHg were considered to be having HTN, which may have led to an overestimation of HTN prevalence in our study. Furthermore, we could not ascertain causality with a cross‐sectional design.

Important risk factors of HTN such as alcohol consumption, smoking habits, physical exercise, hyperglycaemia, waist circumference, and salt consumption were not evaluated in our study. So we could not fully describe the predictors of HTN in our study population.

Despite these limitations, and with the large sample size and robust analytic techniques used in this study, we hope our findings herein reflect to a certain degree the prevalence, awareness, treatment, and control of HTN amongst the elderly in rural Cameroon.

## 5 CONCLUSION

One of 2 elderly in a rural Cameroonian population suffers from HTN, with 6 of 10 of these elderly being aware of their status. Nine of 10 who are aware of their status are on treatment, with only 3 of 10 elderly on treatment having their BP controlled. Nonetheless, emphasis should be on patient education and the promotion of a healthy lifestyle to improve the rates of BP control amongst patients on treatment for HTN. From our study, being obese/overweight is significantly associated with HTN amongst elderly individuals. Evidence‐based interventions such as a reduction in salt intake, increase in fruits and vegetable intake, and frequent physical activity are urgently needed to curb the burden of HTN amongst the elderly in rural Cameroonian communities. We recommend the organization of free consultation days for elderly patients to improve HTN detection and the creation of a HTN clinic in all primary care hospitals to improve treatment and control rates amongst patients with HTN.

## CONFLICT OF INTEREST

The authors report no specific funding in relation to this research and declare no conflicts of interest.

## AUTHOR CONTRIBUTIONS

Conceptualization: Alfred K. Njamnshi, Frank L. Tianyi

Data curation: Valirie N. Agbor

Formal analysis: Valirie N. Agbor

Methodology: Alfred K. Njamnshi, Frank L. Tianyi

Resources: Frank L. Tianyi

Software: Valirie N. Agbor

Supervision: Alfred K. Njamnshi

Writing – original draft preparation: Frank L. Tianyi

Writing – review and editing: Valirie N. Agbor and Alfred K. Njamnshi

All authors have read and approved the final manuscript.
